# Large Language Models for Cardiovascular Disease, Cancer, and Mental Disorders: A Review of Systematic Reviews

**DOI:** 10.3390/healthcare14010045

**Published:** 2025-12-24

**Authors:** Andreas Triantafyllidis, Sofia Segkouli, Stelios Kokkas, Anastasios Alexiadis, Evdoxia Eirini Lithoxoidou, George Manias, Athos Antoniades, Konstantinos Votis, Dimitrios Tzovaras

**Affiliations:** 1 Information Technologies Institute, Centre for Research and Technology Hellas, 57001 Thessaloniki, Greece; sofia@iti.gr (S.S.); s.kokkas@iti.gr (S.K.); talex@iti.gr (A.A.); elithoxo@iti.gr (E.E.L.); tzovaras@iti.gr (D.T.); 2 Department of Digital Systems, University of Piraeus, 18534 Piraeus, Greece; gmanias@unipi.gr; 3 Stremble Ventures Ltd., 4042 Limassol, Cyprus; athos.antoniades@stremble.com

**Keywords:** large language models, generative AI, digital health, literature review

## Abstract

**Background/Objective:** The use of Large Language Models (LLMs) has recently gained significant interest from the research community toward the development and adoption of Generative Artificial Intelligence (GenAI) solutions for healthcare. The present work introduces the first meta-review (i.e., review of systematic reviews) in the field of LLMs for chronic diseases, focusing particularly on cardiovascular, cancer, and mental diseases, to identify their value in patient care, and challenges for their implementation and clinical application. **Methods:** A literature search in the bibliographic databases of PubMed and Scopus was conducted following the Preferred Reporting Items for Systematic Reviews and Meta-Analyses (PRISMA) guidelines, to identify systematic reviews incorporating LLMs. The original studies included in the reviews were synthesized according to their target disease, specific application, LLMs used, data sources, accuracy, and key outcomes. **Results:** The literature search identified 5 systematic reviews respecting our inclusion and exclusion criteria, which examined 81 unique LLM-based solutions. The highest percentage of the solutions targeted mental disease (86%), followed by cancer (7%) and cardiovascular disease (6%), implying a large research focus in mental health. Generative Pre-trained Transformer (GPT)-family models were used most frequently (~55%), followed by Bidirectional Encoder Representations from Transformers (BERT) variants (~40%). Key application areas included depression detection and classification (38%), suicidal ideation detection (7%), question answering based on treatment guidelines and recommendations (7%), and emotion classification (5%). Study aims and designs were highly heterogeneous, and methodological quality was generally moderate with frequent risk-of-bias concerns. Reported performance varied widely across domains and datasets, and many evaluations relied on fictional vignettes or non-representative data, limiting generalisability. The most significant found challenges in the development and evaluation of LLMs include inconsistent accuracy, bias detection and mitigation, model transparency, data privacy, need for continual human oversight, ethical concerns and guidelines, as well as the design and conduction of high-quality studies. **Conclusions:** While LLMs show promise for screening, triage, decision support, and patient education—particularly in mental health—the current literature is descriptive and constrained by data, transparency, and safety gaps. We recommend prioritizing rigorous real-world evaluations, diverse benchmark datasets, bias-auditing, and governance frameworks before LLM clinical deployment and large adoption.

## 1. Introduction

The advent of Large Language Models (LLMs), exemplified by platforms such as ChatGPT, is a significant advancement in Generative Artificial Intelligence (GenAI) with far-reaching implications for improving healthcare [1]. LLMs are able to generate human-like text, answer questions, and complete other language-related tasks with high accuracy [2]. LLMs have demonstrated strong capacity for complex clinical reasoning, patient education, and decision support across a variety of medical fields such as psychiatry [3], cardiology [4], and oncology [5].

**Figure 1:**
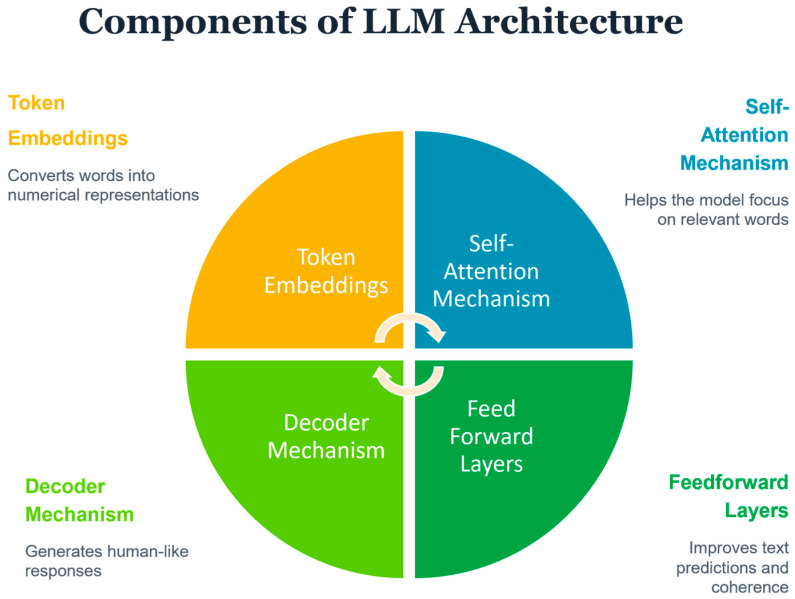
Key components of LLM.

LLMs are artificial intelligence systems built on Transformers [6], an Artificial Neural Network architecture which processes and generates human language by learning statistical patterns from massive amounts of data (e.g., books, websites, scientific papers). At their core, LLMs consist of billions of interconnected parameters (adjustable numerical values) that are trained to predict the next word in a sequence. The key components of an LLM are (Figure 1): (a) Token Embeddings: Converts words into numerical representations for the AI model to process, (b) Self-Attention Mechanism: Helps the model focus on the most relevant words in a sentence, (c) Feedforward Layers: Improves text predictions and sentence coherence, and (d) Decoder Mechanism: Generates human-like responses based on context. The two main types of LLMs used in healthcare are GPT (Generative Pre-trained Transformer) models [7], which excel at generating coherent text for tasks like answering patient questions or drafting clinical summaries [8,9], and BERT (Bidirectional Encoder Representations from Transformers) models [10], which specialize in understanding and classifying existing text for applications like detecting depression from social media posts or identifying suicide risk in clinical notes [11].

The adoption of LLMs in healthcare emerges as a promising step to provide more efficient, safer, and personalized care for patients, mainly because of their capability for swift natural language communication with the users, along with synthesis, summarisation, and contextual reasoning over diverse clinical information, around the clock. The acceptability, usability, and potential of LLMs in improving health and well-being have been explored in several previous studies [12,13,14].

As the body of peer-reviewed research on LLMs in healthcare expands rapidly, there is a pressing need to establish a solid evidence base regarding their clinical effectiveness and practical value. To address this gap, we present a review of systematic reviews examining the use of LLMs among patients with chronic diseases—with particular emphasis on cardiovascular, oncological, and mental health conditions because of their significant global burden. To the authors’ knowledge, this meta-review is the first in the field of LLMs for healthcare.

Our work aims to analyze the characteristics, performance, benefits and methodological challenges of LLM-based interventions and tools, in this fast-evolving domain. Unlike disease-specific analyses, the current review adopts a cross-condition perspective, enabling a broader understanding of how LLMs are being leveraged across diverse chronic care contexts. By systematically mapping the current state of applications, evaluating their effectiveness, and highlighting current challenges, this study seeks to inform researchers, developers, clinicians, and policymakers in designing and implementing more robust and impactful LLM-driven health interventions, towards realizing the full potential of GenAI application in clinical practice.

## 2. Methodology

We searched the bibliographic databases of PubMed and Scopus to identify systematic reviews of the application of LLMs for cardiovascular disease, cancer, and mental disorders, as reported in manuscripts published after 2022, since this was the year in which the interest in LLMs exploded because of the landmark emergence of ChatGPT. The inclusion criteria were: (a) the review should be defined as systematic, focus on the effectiveness of the LLMs, and follow reporting guidelines such as the Preferred Reporting Items for Systematic review and Meta-Analyses (PRISMA) [15] or the Cochrane guidelines [16]; (b) the review should report LLM-based tools or interventions targeted at chronically ill individuals diagnosed with cardiovascular disease, cancer, or mental disorders; (c) the paper should be written in English. We used the keyword query (“LLM” OR “large language model” OR “chatbot” OR “conversational” OR “bot” OR “digital assistant” OR “virtual assistant” OR “digital agent” OR “virtual agent”) for search within the title, abstract and keywords of the manuscripts, and restricted the search to a review type of articles. Reviews examining interventions or tools which were not focused on leveraging LLMs were excluded. Furthermore, reviews focusing exclusively on other medical fields such as surgery or medical education were excluded. Reviews not examining quantitative outcomes, surveys, and protocol papers were also excluded from the review.

The selection and review of papers were conducted independently by four reviewers (authors AT, SS, AA, SK) to ensure relevance based on predefined inclusion and exclusion criteria and to minimize potential selection bias or errors. Following the literature search, both abstracts and full-text manuscripts were screened. Studies that did not meet the inclusion criteria were excluded, and only those for which consensus among all reviewers was achieved were retained.

The methodological quality of the included systematic reviews was evaluated using the second version of the AMSTAR tool (A Measurement Tool to Assess Systematic Reviews), which has demonstrated reliability [17]. Data from the primary studies included within these reviews were synthesized (by AT) according to several dimensions: Target disease, application area, LLM(s) used, leveraged data sources, model accuracy, and key outcomes. We report descriptive percentages because most studies provided only aggregated summaries and heterogeneity in outcomes and timing precluded pooling or more advanced meta-analytic techniques.

## 3. Results

### 3.1. Literature Search Outcomes

**Figure 2:**
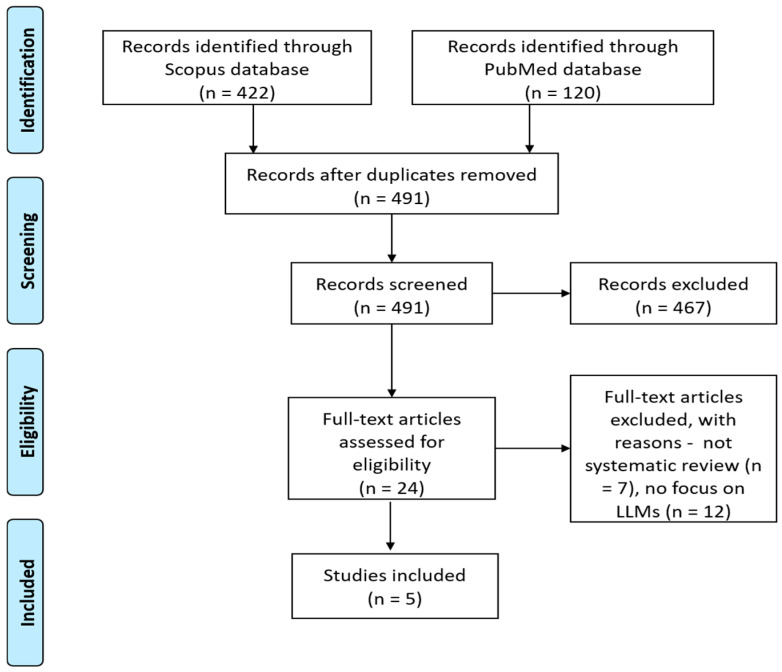
PRISMA flow diagram for study inclusion.

Our literature search in the PubMed and Scopus databases was conducted on November 2024 with the last search update taking place on May 2025, to identify relevant studies published since 2022. The search yielded 422 records from the Scopus database and 120 records from PubMed. After removing all duplicates in the Mendeley© bibliography management software [18], and applying our inclusion and exclusion criteria, 24 articles remained for full manuscript reading. Finally, 5 papers (systematic reviews) were included [19,20,21,22,23]. Reasons for paper exclusion are shown in Figure 2, which is a standard PRISMA flow diagram intended to document the process of study identification, screening, eligibility assessment, and inclusion.

### 3.2. Quality Assessment of Reviews and Original Studies

The quality of the included review studies as assessed through AMSTAR 2 criteria can be seen in Table 1. All reviews performed study selection in duplicate and discussed the heterogeneity of studies and results. However, AMSTAR 2 assessment revealed recurring methodological weaknesses across the included systematic reviews. The most frequently failed domains were: (i) lack of comprehensive search strategies—none of the reviews searched gray literature or consulted experts; (ii) absence of explicit PICO framing in research questions; (iii) insufficient justification for deviations from protocols; and (iv) limited assessment and consideration of risk of bias when interpreting results. Furthermore, most reviews did not report sources of funding for included studies and did not explain the rationale for study design selection. These gaps underscore the need for future systematic reviews in this field to adopt rigorous protocols, transparent reporting, and standardized bias assessment tools to enhance reliability and reproducibility.

Three reviews [20,22,23], assessed the risk of bias of the individual studies using tools such as Quality Assessment of Diagnostic Accuracy Studies (QUADAS 2), Risk of Bias 2 Tool, Risk Of Bias In Non-randomized Studies-of Interventions (ROBINS-I), and Prediction model Risk Of Bias Assessment Tool (PROBAST). The individual studies were reported to be of high risk of bias except one in the review for breast cancer management by Sorin et al. [20], while Guo et al. [22] have reported overall a low risk of bias in the studies for mental health applications. Omar and Levkovich [23] in their review focusing on depression, reported an overall mixed picture in terms of risk of bias assessment. Although most studies were reported to be of low risk of bias in measurements and outcomes, several studies presented moderate biases because of confounding factors and participant selection that may have affected the applicability of the results.

### 3.3. Characteristics of Individual Studies

**Figure 3:**
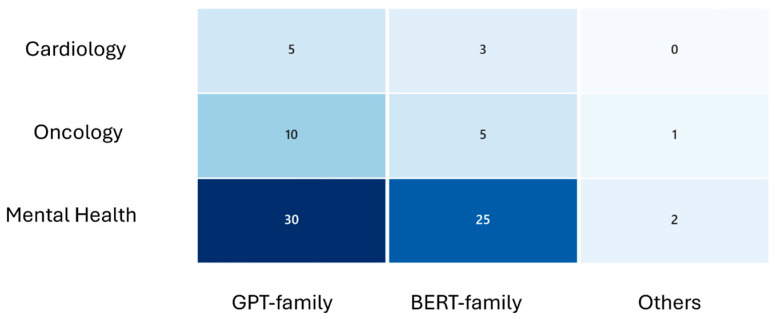
Heatmap of LLM type usage by medical domain (darker blue corresponds to higher frequency).

**Figure 4:**
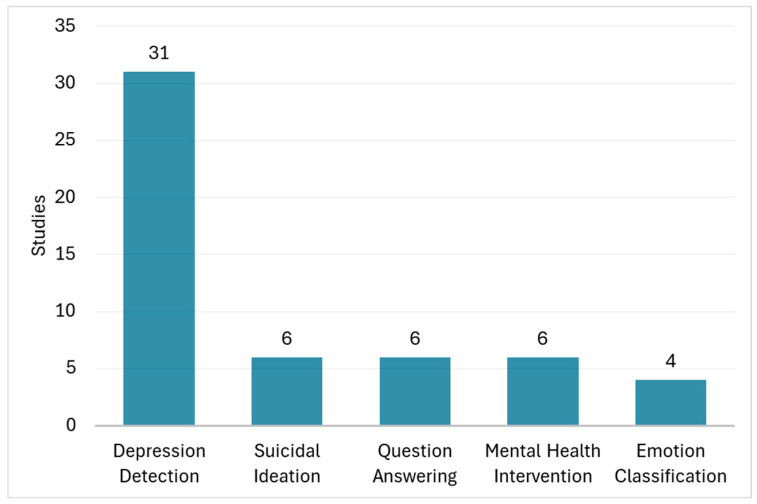
Most common LLM application areas.

In Table A1, the main characteristics of the 81 non-duplicate individual studies reported in the reviews are presented in terms of target disease, specific application, used LLMs, data sources, accuracy, and key outcomes. The majority of the solutions focused on mental health conditions (70 studies, 86%), followed by cancer (6 studies, 7%) and cardiovascular diseases (5 studies, 6%). Most studies employed GPT models (45 studies, 55%), while BERT model and its variants were used in 33 studies (40%) (Figure 3). The main application areas included depression detection and classification (31 studies, 38%), suicidal ideation detection (6 studies, 7%), question answering based on treatment guidelines and recommendations (6 studies, 7%), mental health intervention, e.g., support for loneliness (6 studies, 7%), and emotion classification (4 studies, 5%) (Figure 4). The studies showed substantial heterogeneity: about half (41 studies, 50%) concentrated on clinical or diagnostic accuracy—evaluating correct diagnosis, guideline alignment, or model performance—while others (34 studies, 42%) were descriptive, focusing on aspects such as narrative outcomes, usability, trust, or plausibility.

### 3.4. Summary of LLMs Performance

The LLMs in depression applications (31 studies) achieved an accuracy in detecting the disease or its symptoms from 50% to 97% and an F1 score from 0.42 to 0.93. The studies reporting the highest performance used BERT, RoBERTa, AudiBERT, DistilBERT, and DeBERTa, and leveraged diverse datasets such as tweets, clinical interviews, or the mental health corpus database. The suicidal ideation studies (6 studies) used LLMs such as BERT, GPT, and XLNet, using social media posts (3 studies), as well as fictional patient data (3 studies). The reported achieved accuracy in 3 studies for suicidal ideation detection was from 87% to 95%. LLMs were found also to demonstrate good performance in several other mental health applications, such as classification of psychiatric conditions with BERT (F1 0.83) [24], mental health disorder prediction with BERT through Twitter (accuracy 97%) [25], and sentiment analysis through social media texts using GPT and Open Pre-trained Transformers (accuracy 86%) [26]. However, on other few occasions, LLMs did not demonstrate an acceptable performance, as in the case of the identification of diagnostic and management strategies in psychiatric conditions with GPT (accuracy 61%) [27]. All 6 studies reporting outcomes of diverse mental health interventions, e.g., support for loneliness, mindfulness, and self-discovery, did not provide quantitative performance metrics, however they highlighted the usefulness of LLMs in mental health support, with only one study criticizing the insufficiency of GPT in complex mental health scenarios.

In cardiology applications (5 studies), the LLMs were based on GPT, and they were used for answering clinical questions. The evaluation of the performance was descriptive in most of the studies (3 studies), while an accuracy up to 64.5% was reported in the studies showing performance metrics (2 studies).

In cancer applications (6 studies), GPT models were also used. 3 studies focused on tumor board clinical decision support, 2 studies on question-answering, and 1 study assessed the time and cost for developing LLM prompts. The accuracy of LLM responses compared with reviews of the tumor board varied, reaching up to 70% with real patient data, and up to 95% with fictional patient data. The question-answering applications reached up to 98% accuracy. Regarding time and cost evaluation, LLM-based prompting was found to offer an efficient approach to extract key information from the medical records of breast cancer patients and to generate well-structured clinical datasets. This method is expected to significantly reduce the effort required in routine clinical practice and research.

### 3.5. Benefits of Using LLMs

Across mental health, oncology, and cardiology, the most consistently reported benefits of LLMs include early detection and screening capabilities, which dominate mental health applications and are emerging in oncology through pathology report parsing. Decision-support functions are common to all three domains, but oncology demonstrates the highest complexity in multidisciplinary planning, while cardiology emphasizes guideline-concordant question answering. Patient education and engagement appear universally beneficial, with mental health leveraging conversational empathy and oncology focusing on informed consent. Documentation and research support are reported across all domains, but oncology highlights efficiency gains in tumor board preparation, whereas cardiology emphasizes educational utility.

#### 3.5.1. Detection and Screening

LLMs have shown significant potential in the improvement of detection and screening for diseases. In mental health, transformer-trained LLMs have been applied to detect depression, suicidality, and anxiety, based on the examination of linguistic features and affectual cues within patient-written text or social media posts [21,22]. This strategy enables the early identification of mental illness within non-clinical environments, with the provision of scalable non-intrusive screening tools that can be used to monitor public health. The ability of LLMs to process large volumes of unstructured language data allows for timely detection of subtle indicators of distress that may escape conventional screening tools.

In oncology, benefits of screening procedures are also apparent. LLMs can extract tumor, receptor, and staging relevant variables from pathology as well as imaging reports with a high accuracy, reaching up to 98% [20]. This not only fastens the data-processing time but also helps standardized diagnostic workflows through the reduction in human failure as well as enhanced information-extraction consistency. Taken together, these outcomes establish that LLMs could bridge the gap between raw clinical data to usable diagnostic information to facilitate earlier treatment as well as personalized care.

#### 3.5.2. Risk Assessment and Triage

In addition to screening and detection, LLMs enable risk assessment and triage procedures, especially in mental health applications. By analyzing the sentiment, tone, and complexity of language, such models can evaluate the psychological distress and guide those with increased risk of suicide or crisis [23]. This feature is particularly useful in online or resource-limited care environments, where fast triage can easily affect clinical outcomes.

The analytical power of the LLMs also helps practitioners in correlating large amounts of patient-reported information to assist with triage decisions regarding who needs immediate intervention as opposed to ongoing monitoring. As the systems evolve, the integration of the triage tools with the telepsychiatry platforms could allow long-term, passive surveillance of mental state with earlier detection of mental health emergencies.

#### 3.5.3. Clinical Reasoning and Decision Support

The LLMs showed increasing abilities to mimic clinical reasoning and facilitate decision-making within psychiatry, cardiology, and oncology. They can enable diagnosis and treatment planning in psychiatry, typically producing guideline-concordant reasoning when presenting with clinical vignettes [21,22]. This helps the clinician in challenging diagnostic situations but also provides a useful instrument within medical education, as it enables students to practice structured ways of reasoning.

In cardiology, the LLMs proved effective in accurately answering board-style examination questions and depicting clinical reasoning in case discussions [4]. Such functions advance both educational use and ongoing professional development with the option to present an adaptive learning environment that resembles clinician-level judgment.

In oncology, the LLMs aid in the synthesis of multidisciplinary cases and tumor board planning with 50–70% agreement with the expert panels [5]. This analytical potential, in turn, leads to the possibility of summarizing variable clinical facts and helping with decision-making processes, most specifically where combined therapies need to be incorporated.

#### 3.5.4. Patient Education and Participation

A major advantage of LLMs is their ability to encourage patient engagement by using natural, conversational interfaces. In mental health, conversational agents that use LLMs can provide psychoeducation, empathetic dialog, and stigma reduction by offering support to individuals who might otherwise avoid seeking for help [2]. These systems can deliver round-the-clock guidance and emotional validation, contributing to improved accessibility and continuity of care. In cardiology, the LLMs were shown to produce accurate and sympathetic responses to patient questions, enhancing compliance with regimens as well as health literacy [4]. Similarly, in oncology, they enable the development of patient education materials and consent summaries incorporating the most current clinical practice guidelines [5]. Such functionalities endow the patient with reliable, easy-to-read information, enforcing shared decision-making as well as faith in communication with the clinician.

#### 3.5.5. Summary, Documentation, and Research Support

In all areas, LLMs bring significant advantages in automating research and documentation procedures. They help in psychiatry to summarize transcripts of therapy and to produce structured sets of data for Natural Language Processing (NLP) studies, minimizing manual labor and maximizing analytical potential [22]. They help in cardiology and oncology to prepare discharge summaries, clinical notes, and guideline overviews to minimize administrative workload [19,20]. In addition, the models aid clinical researchers in scientific paper writing through the automation of evidence synthesis and paper composition [19]. This aspect can accelerate the publication of clinical evidence, boosting the efficiency of medical scholarship. As the science of LLMs advances, this integration into research workflows could revolutionize the creation, curation, and communication of medical knowledge.

### 3.6. Challenges of Using LLMs

The most critical challenges for real-world adoption of LLMs are consistent across domains: hallucinations and output correctness remain universal concerns, particularly acute in oncology where treatment decisions carry high stakes. Bias and transparency issues are pervasive, with mental health applications most vulnerable to demographic and linguistic skew due to reliance on social media data. Data privacy and regulatory compliance challenges are emphasized in oncology and cardiology because of sensitive clinical records. Continuous human oversight is unanimously recommended, but mental health reviews stress its necessity for emergency scenarios, while cardiology and oncology focus on diagnostic validation. Methodological rigor and lack of real-world trials are cross-cutting limitations, hindering generalizability and safe deployment.

#### 3.6.1. Accuracy, Safety, and Efficacy of LLMs in Real World Settings

Although LLMs showed better results than traditional tools such as machine learning [28,29], and even exhibited capabilities comparable to those of human experts in some cases [30,31], variations in accuracy and output correctness across different tasks persist. As an example, Levkovich and Elyoseph [32], evaluated ChatGPT’s performance in assessing suicide risk highlighting that ChatGPT could underestimate or overestimate suicide risks compared to mental health professionals, especially in complex scenarios with high perceived burdensomeness and thwarted belongingness. Advanced models such as GPT-4 have been effective in interpreting clinical and unstructured data to manage, detect, and classify depression [33]. However, studies often rely on fictional clinical vignettes, limiting the generalizability of these findings in real-world clinical practice [34]. In this context, researchers and industrial stakeholders should gather enough evidence of efficacy and safety—through rigorous clinical studies, testing, and oversight—before deploying LLMs at scale in real-life, to prevent causing harm due to unverified performance.

All reviews underscore the enduring risk of hallucinations, wherein models generate information that is incorrect, incomplete, or entirely fabricated. Such outputs—ranging from inaccurate facts to invented citations—pose danger in clinical settings, where they may inadvertently mislead healthcare professionals or patients. The reviews consistently identify hallucination control as a fundamental technical challenge that must be addressed prior to any autonomous clinical deployment of LLMs.

#### 3.6.2. Bias and Model Transparency

Training data biases (demographic, geographic, linguistic) may propagate into LLM outputs. Several reviews note age, language and population skews (e.g., social-media datasets over-represent younger, English-speaking users), leading to uneven performance and potential amplification of disparities in care. Detecting and mitigating bias is made harder because training corpora and curation processes for commercial models are often undisclosed. Reviews call for benchmark datasets with diverse, annotated examples and for bias-auditing pipelines. Ensuring demographic inclusivity, representational diversity, and transparency in LLM development is essential to safeguard public trust in AI-driven healthcare systems.

A critical assessment of the safety and trust of LLMs was conducted in included review studies, highlighting the “black box” nature of AI systems. This means that LLMs are associated with limited interpretability and transparency (why the model generated a given answer), which undermines trust and makes clinical validation and regulatory assessment challenging. The reviews recommend model documentation, provenance of training data, auditing, and research into explanatory methods (attention analyses, knowledge graphs, causal embeddings), to improve model transparency.

#### 3.6.3. Data Privacy, Security, and Regulatory Challenges

All reviews identify significant challenges related to data protection and regulatory compliance when applying LLMs in healthcare. Cloud-based model architectures risk exposing sensitive health information to third-party servers, while conventional anonymization is often inadequate, as models may inadvertently reconstruct or reveal personal data. The literature highlights the need for privacy-preserving solutions such as federated learning, encrypted inference, and local on-premise deployments. Regulatory frameworks—including the GDPR and the forthcoming EU AI Act—remain ill-equipped to address GenAI, leaving uncertainties over accountability, liability, and data-control roles. The opacity of commercial models further complicates auditability and certification under existing medical device standards. Reviews therefore call for transparent data-governance mechanisms, regulatory sandboxes, and privacy-by-design principles to ensure both ethical integrity and legal compliance. Ultimately, safeguarding patient privacy is viewed as a prerequisite for the responsible clinical integration of LLMs.

#### 3.6.4. Data Availability and Generalizability

Data scarcity and imbalance have been identified as core barriers to reliable LLM performance in healthcare. Most models are trained or tested on English-language, high-resource datasets—such as PubMed abstracts or online social media—rather than representative clinical data, limiting generalizability across cultures, age groups, and care settings. Under-representation of non-English, minority, and low-income populations leads to systematic performance gaps and potential inequities in care. The opacity of proprietary training corpora further restricts reproducibility and independent bias auditing, raising concerns about compliance with ethical and data protection standards. To address these gaps, the reviews call for open, multilingual benchmark datasets annotated by domain experts, and privacy-preserving data sharing approaches. Strengthening data diversity, transparency, and accessibility is seen as essential to ensure that LLMs achieve equitable, safe, and scientifically valid integration into healthcare practice.

#### 3.6.5. Continuous Human Oversight and Ethical Governance Frameworks

All five reviews converge on the principle that large language models must operate under continuous human supervision when applied in clinical or mental health contexts. While LLMs demonstrate promise in tasks such as information retrieval, triage support, and patient education, their susceptibility to hallucinations, bias, and contextual misinterpretation makes their unsupervised use unsafe. The reviews emphasize that LLMs should function as decision-support tools rather than autonomous agents, complementing but not replacing expert judgment. Ongoing human monitoring is also crucial for detecting subtle model drift or unexpected behavior following software updates. In this direction, structured human-in-the-loop frameworks, with clearly defined escalation procedures for high-risk outputs (e.g., suicide ideation detection, diagnostic recommendations) are required [35].

All five reviews stress that deploying LLMs in healthcare demands clear ethical governance frameworks ensuring transparency, accountability, and respect for patient rights. Current practice often outpaces regulation, leaving uncertainty around responsibility, informed consent, and fairness. Reviews highlight core principles such as accountability (clinicians retain final responsibility), transparency (users must know when AI is involved), non-maleficence (preventing harm through validation), justice (equitable performance across populations), autonomy (informed consent for AI interaction), and data ethics (responsible stewardship and privacy protection). Overall, the literature emphasizes that robust, enforceable ethical frameworks—combining technical, institutional, and societal safeguards—are essential to maintain public trust and ensure that GenAI serves healthcare’s fundamental moral obligations.

#### 3.6.6. Methodological Quality of LLM Studies

All reviews highlight significant methodological weaknesses in current research on LLMs in healthcare. Most studies rely on retrospective, simulated, or vignette-based data rather than real-world clinical trials, limiting external validity and generalizability. Evaluation protocols vary widely, with inconsistent reporting of datasets, prompts, metrics, and baseline comparisons, making cross-study synthesis difficult. Few investigations employ standardized bias or risk-of-bias tools (e.g., QUADAS-2, PROBAST), and many omit details about model versioning or update cycles—critical for reproducibility. Reviews call for prospective, pre-registered, and multi-institutional studies using transparent methods and benchmark datasets that reflect clinical complexity and population diversity. They also emphasize the need for longitudinal evaluations assessing safety, performance drift, and human–AI interaction over time. Overall, improving methodological rigor and transparency is seen as essential to move the field from proof-of-concept experimentation toward clinically validated, ethically sound, and policy-relevant evidence. A thematic synthesis of outcomes and challenges for LLMs covering mental health, oncology, and cardiology applications can be seen in Table 2.

## 4. Discussion

### 4.1. Main Findings

We conducted a review of systematic reviews on the application of LLMs in critical healthcare domains, including cardiology, oncology, and mental health. The main goal was to examine the characteristics, outcomes, and challenges of LLMs by drawing, exploring, and synthesizing the findings of systematic reviews in this novel area of research. The key finding of the review is that LLMs have emerged as potential enhancers of diagnostic and decision-support processes in cardiology, oncology, and mental health. Nonetheless, the limited rigorous evidence to date underscores the need for robust, real-world research to validate LLM effectiveness and safety in clinical practice.

The predominant research focus was on depression detection and classification, complemented by investigations into suicidal ideation detection, question answering aligned with clinical guidelines, AI-driven mental health interventions such as loneliness support, emotion recognition and classification, and tumor board clinical decision support. More than half of the included studies applied GPT-based models, while BERT and its derivatives were employed in approximately 40% of the studies. The performance of LLMs was variable across their broad spectrum of applications, and therefore no clear evidence of their effectiveness can be demonstrated currently.

Across cardiology, oncology, and mental health, the reviewed evidence indicates that LLMs have substantial potential to enhance various aspects of clinical care, education, and research. Their strongest demonstrated benefits lie in detection, triage, and decision support, where transformer-based architectures can process vast volumes of unstructured text to identify early markers of disease, assess patient risk, and generate structured diagnostic information with high accuracy.

In mental health, LLMs have been applied to detect depression, anxiety, and suicidality through linguistic and affective cues, enabling scalable, non-intrusive screening beyond traditional clinical environments. In this context, LLMs may facilitate risk stratification and triage, helping clinicians identify individuals in need of early intervention—particularly in resource-limited or telehealth settings [36]. Their capacity for clinical reasoning and decision support has been demonstrated also in cardiology and oncology, where models often produce guideline-concordant responses and may assist in diagnostic synthesis and multidisciplinary planning [37].

Another prominent advantage of LLMs is their ability to enhance patient education and engagement through empathetic, conversational interfaces that provide accessible health information and encourage adherence and shared decision-making [38]. Additionally, LLMs streamline documentation, summarization, and research workflows, reducing administrative burden and accelerating knowledge generation. By automating report generation, the literature synthesis, and evidence summarisation, they can increase efficiency across clinical research domains [39].

Despite the potential of LLMs in improving clinical care, several limitations and challenges toward their wide adoption can be highlighted. Model output correctness and hallucinations remain fundamental challenges in applying LLMs to healthcare. Models frequently produce plausible but inaccurate or fabricated information, including erroneous clinical facts or nonexistent references [40]. Such hallucinations undermine reliability and pose safety risks when used for diagnosis or patient communication. The literature attributes these errors to limitations in the inherent logical structure of LLMs, training data, probabilistic text generation, and lack of factual grounding.

Furthermore, the included reviews consistently highlighted bias and lack of transparency as critical obstacles to the safe deployment of LLMs in healthcare. In several cases, the opacity of proprietary model architectures and datasets prevents independent auditing, bias detection, and accountability [41]. In this direction, transparent reporting of data provenance, inclusive and diverse training corpora, and bias-auditing frameworks that systematically evaluate fairness and performance across demographic and linguistic groups before clinical implementation, are important steps toward the improvement of LLMs’ reliability.

The reviewed evidence underscores major challenges related to data privacy, availability, human oversight, and methodological rigor in LLM research. Most models rely on proprietary or web-scraped data with uncertain consent and provenance, raising privacy and compliance concerns under frameworks such as GDPR. Simultaneously, the lack of open, diverse, expert-labeled, and representative datasets limits reproducibility and generalizability across languages and populations. The reviews stress that continuous human oversight is indispensable—LLMs should function as decision-support tools, with clinicians validating outputs and managing escalation for high-risk content. Methodologically, the current literature remains exploratory, dominated by simulated or retrospective designs with inconsistent evaluation metrics and scarce real-world validation. To advance clinical integration responsibly, the field must prioritize transparent data governance, ethical data sharing, standardized evaluation protocols, and prospective trials that rigorously test performance, safety, and reliability in real-world healthcare settings. Ensuring computational efficiency is also essential for real-time clinical applications, as models must deliver accurate outputs with minimal latency to support tasks like triage, decision support, and patient communication without disrupting care workflows.

The findings of this meta-review have important implications for the future of AI-driven healthcare. By synthesizing evidence across cardiology, oncology, and mental health, this work highlights that LLMs have the potential to transform clinical workflows through language-driven intelligence. Their demonstrated benefits in early detection, triage, decision support, and patient engagement suggest that LLMs could significantly improve clinical decision making and enable more personalized care delivery. At the same time, the variability in performance and methodological gaps underscore the need for rigorous validation before widespread adoption. These insights call for a strategic focus on developing transparent, bias-resistant, and ethically governed LLM systems, ensuring that innovation translates into safe, equitable, and clinically meaningful outcomes. Ultimately, the integration of LLMs into healthcare could redefine how clinicians interact with data, patients, and decision-making processes, accelerating the transition toward more efficient and patient-centered care.

### 4.2. Limitations

This review should be interpreted within the context of its limitations. The authors used a limited set of terms for the search of literature, which might have resulted in the omission of other relevant studies. Our search design likely missed relevant reviews that used terms referring to specific health conditions, alternative AI terminology, or did not mention the AI tool specifically in those fields. We limited our literature search to PubMed and Scopus, which are widely used databases internationally. This choice, however, represents an important methodological constraint that may introduce selection bias and affect the conclusiveness of our synthesis. We also recommend that future updates of this review expand the search to additional databases such as Embase and Cochrane, which are likely to index systematic reviews with rigorous clinical trial data. The review was based on the findings from systematic reviews of LLM studies and different populations in terms of disease, age, education and socioeconomic level were studied in different settings, which prevented the conduction of meta-analysis. The limitations of the included systematic reviews (introduced, for example, in their inclusion and exclusion criteria) might have affected the representation of the progress of LLMs. This review presented characteristics and outcomes of LLMs for cardiovascular disease, cancer, and mental disorders, considering their global burden. LLMs for other chronic conditions such as diabetes, arthritis, or liver disease were not examined. Our literature search was restricted to reviews published after 2022, due to the landmark emergence of ChatGPT. Different results could emerge if older studies were also included. Most included studies examined by the included reviews relied on controlled or simulated datasets rather than real-world clinical data, which limits the generalizability of findings and underscores the need for prospective evaluations in diverse, real-world settings. The generalizability of the findings is further restricted by the fact that only a small number of studies were found to be eligible for inclusion in this review.

## 5. Conclusions

In conclusion, the collective evidence from recent systematic reviews demonstrates that LLMs hold substantial promise for enhancing healthcare through improved detection, triage, decision support, and patient engagement. Overall, the reviews converge on the view that while LLMs are not yet ready for autonomous deployment in clinical settings, their augmentation of human expertise—from early detection and clinical reasoning to patient communication and research support—positions them as valuable tools for improving efficiency, consistency, and accessibility of healthcare services. Yet, their clinical integration remains constrained by persistent challenges, including hallucinations, bias, lack of transparency, data privacy risks, and limited methodological robustness. Current research is largely exploratory, emphasizing the need for rigorous, real-world evaluations supported by transparent data governance and ethical oversight. Ensuring reliability, fairness, and human supervision will be essential to build trust and safeguard patient welfare. As these models evolve, interdisciplinary collaboration among clinicians, data scientists, ethicists, and policymakers will be crucial to translating technical innovation into safe, equitable, and accountable clinical practice. Properly governed and validated, LLMs could become transformative instruments in shaping the future of evidence-based, human-centered healthcare.

## Figures and Tables

**Table 1:** Quality assessment of the included reviews according to AMSTAR 2 criteria (Y: Yes, N: No, PY: Partial Yes) of included studies (Original items concerning meta-analysis and quantitative synthesis were removed because they were deemed to be out of scope).

AMSTAR 2 Criteria	Study				
	Sharma et al. [19]	Sorin et al. [20]	Omar et al. [21]	Guo et al. [22]	Omar and Levkovich [23]
1. Did the research questions and inclusion criteria for the review include the components of PICO?	N	N	N	N	N
2. Did the report of the review contain an explicit statement that the review methods were established prior to the conduct of the review and did the report justify any significant deviations from the protocol?	N	N	PY	PY	PY
3. Did the review authors explain their selection of the study designs for inclusion in the review?	N	N	N	N	N
4. Did the review authors use a comprehensive literature search strategy?	PY	N	PY	PY	PY
5. Did the review authors perform study selection in duplicate?	Y	Y	Y	Y	Y
6. Did the review authors perform data extraction in duplicate?	Y	N	Y	Y	Y
7. Did the review authors provide a list of excluded studies and justify the exclusions?	N	PY	N	Y	PY
8. Did the review authors describe the included studies in adequate detail?	N	N	PY	PY	PY
9. Did the review authors use a satisfactory technique for assessing the risk of bias (RoB) in individual studies that were included in the review?	N	Y	N	Y	Y
10. Did the review authors report on the sources of funding for the studies included in the review?	N	N	N	N	N
11. Did the review authors account for RoB in individual studies when interpreting/discussing the results of the review?	N	N	N	N	N
12. Did the review authors provide a satisfactory explanation for, and discussion of, any heterogeneity observed in the results of the review?	Y	Y	Y	Y	Y
13. Did the review authors report any potential sources of conflict of interest, including any funding they received for conducting the review?	Y	Y	Y	Y	Y

**Table 2:** Thematic synthesis of LLM outcomes and challenges.

Category	Detailed Summary
Clinical Applications	- Mental Health: Depression detection, suicidality risk assessment, emotion classification.
	- Oncology: Tumor board decision support, pathology report parsing, question answering.
	- Cardiology: Guideline-based question answering, diagnostic reasoning, triage support.
Patient Engagement	- Mental Health: Conversational agents for psychoeducation, stigma reduction, loneliness support.
	- Oncology: Consent preparation, patient education materials.
	- Cardiology: Adherence support, health literacy improvement.
Technical Challenges	- Accuracy variability across tasks and domains.
	- Hallucinations and fabricated outputs.
	- Bias in training data (age, language, geography).
	- Lack of interpretability and transparency.
Ethical & Regulatory Issues	- Data privacy risks in cloud-based deployments.
	- Compliance with GDPR and emerging AI regulations.
	- Accountability and liability uncertainties.
	- Need for human-in-the-loop governance.
Methodological Gaps	- Heavy reliance on simulated or vignette-based data.
	- Scarcity of prospective, real-world trials.
	- Inconsistent evaluation metrics and reporting standards.
	- Limited demographic and linguistic diversity in datasets.

## Data Availability

No new data were created or analyzed in this study.

## Appendix A

**Table A1:** Characteristics of individual studies included in the reviews.

Study	Target Disease	Application	LLM Used	Data Sources	Accuracy	Key Outcomes
Kusunose et al. [42]	CVD	Question-answering based on Japanese Hypertension guidelines	GPT 3.5	Japanese Society of Hypertension Guidelines for the Management of Hypertension	64.50%	ChatGPT performed well on clinical questions, performance on hypertension treatment guidelines topics was less satisfactory
Rizwan et al. [43]	CVD	Question-answering on treatment and management plans	GPT 4.0	Hypothetical questions simulating clinical consultation	N/A	Out of the 10 clinical scenarios inserted in ChatGPT, eight were perfectly diagnosed
Skalidis et al. [44]	CVD	Ability in European Cardiology exams	GPT (Version N/A)	Exam questions from the ESC website, StudyPRN and Braunwald’s Heart Disease Review and Assessment	58.80%	Results demonstrate that ChatGPT succeeds in the European Cardiology exams
Van Bulck et al. [45]	CVD	Question-answering on common cardiovascular diseases	GPT (Version N/A)	Virtual patient questions	N/A	Experts considered ChatGPT-generated responses trustworthy and valuable, with few considering them dangerous. Forty percent of the experts found ChatGPT responses more valuable than Google
Williams et al. [46]	CVD	Question-answering on cardiovascular computed tomography	GPT 3.5	Questions from the Society of Cardiovascular Computed Tomography 2023 program as well as questions about high risk plaque (HRP), quantitative plaque analysis, and how AI will transform cardiovascular CT	N/A	The answers to debate questions were plausible and provided reasonable summaries of the key debate points
Choi et al. [47]	Breast Cancer	Evaluation of the time and cost of developing prompts using LLMs, tailored to extract clinical factors in breast cancer patients	GPT 3.5	Data from reports of surgical pathology and ultrasound from 2931 breast cancer patients who underwent radiotherapy from 2020 to 2022	87.70%	Developing and processing the prompts took 3.5 h and 15 min, respectively. Utilizing the ChatGPT application programming interface cost US $65.8 and when factoring in the estimated wage, the total cost was US $95.4
Griewing et al. [48]	Breast Cancer	Concordance to tumor board clinical decisions	GPT 3.5	Fictious patient data with clinical and diagnostic data	50−95%	ChatGPT 3.5 can provide treatment recommendations for breast cancer patients that are consistent with multidisciplinary tumor board decision making of a gynecologic oncology center in Germany
Haver et al. [49]	Breast Cancer	Question-answering on breast cancer prevention and screening	GPT 3.5	25 questions addressing fundamental concepts related to breast cancer prevention and screening	88%	ChatGPT provided appropriate responses for most questions posed about breast cancer prevention and screening, as assessed by fellowship-trained breast radiologists
Lukac et al. [50]	Breast Cancer	Tumor board clinical decision support	GPT 3.5	Tumor characteristics and age of the 10 consecutive pretreatment patient cases	64.20%	ChatGPT provided mostly general answers based on inputs, generally in agreement with the decision of MDT
Rao et al. [51]	Breast Cancer	Question-answering based on American Collegy of Radilology recommendations	GPT 4.0, GPT 3.5	Prompting mechanisms and clinical presentations based on American College of Radiology	88.9−98.4%	ChatGPT displays impressive accuracy in identifying appropriateness of common imaging modalities for breast cancer screening and breast pain
Sorin et al. [52]	Breast Cancer	Tumor board clinical decision support	GPT 3.5	Clinical and diagnostic data of 10 patients	70%	Chatbot’s clinical recommendations were in-line with those of the tumor board in 70% of cases
Abilkaiyrkyzy et al. [53]	Mental Disease	Mental illness detection using a chatbot	BERT	219 E-DAIC participants	69%	Chatbot effectively detects and classifies mental health issues, highly usable for reducing barriers to mental health care
Adarsh et al. [54]	Mental Disease	Depression sign detection using BERT	BERT-small	Social media texts	63.60%	Enhanced BERT model accurately classifies depression severity from social media texts, understanding nuances better than others
Alessa and Al-Khalifa [55]	Mental Disease	Mental health interventions using CAs for the elderly supported by LLMs	ChatGPT, Google Cloud API	Record of interactions with CA; results of the human experts’ assessment	N/A	The proposed ChatGPT-based system effectively serves as a companion for elderly individuals, helping to alleviate loneliness and social isolation. Preliminary evaluations showed that the system could generate relevant responses tailored to elderly personas
Beredo and Ong [56]	Mental Disease	Mental health interventions using CAs supported by LLMs	EREN, MHBot, PERMA	Empatheticdialogues (24,850 conversations); Well-Being Conversations; Perma Lexica	N/A	This study successfully demonstrated a hybrid conversation model, which combines generative and retrieval approaches to improve language fluency and empathetic response generation in chatbots. This model, tested through both automated metrics and human evaluation, showed that the medium variation in the FTER model outperformed the vanilla DialoGPT in perplexity and that the human-likeness, relevance, and empathetic qualities of responses were significantly enhanced, making VHope a more competent CA with empathetic abilities
Berrezueta-Guzman et al. [57]	Mental Disease	Evaluation of the efficacy of ChatGPT in mental intensive treatment	ChatGPT	Evaluations from 10 attention deficit hyperactivity disorder (ADHD) therapy experts and interactions between therapists and the custom ChatGPT	N/A	This paper found that the custom ChatGPT demonstrated strong capabilities in engaging language use, maintaining interest, promoting active participation, and fostering a positive atmosphere in ADHD therapy sessions, with high ratings in communication and language. However, areas needing improvement were identified, particularly in confidentiality and privacy, cultural and sensory sensitivity, and handling nonverbal cues
Blease et al. [58]	Mental Disease	Evaluation of psychiatrists’ perceptions of the LLMs	ChatGPT, Bard, Bing AI	Survey responses from 138 APA members on LLM chatbot use in psychiatry	N/A	This paper found that over half of psychiatrists used AI tools like ChatGPT for clinical questions, with nearly 70% agreeing on improved documentation efficiency and almost 90% indicating a need for more training while expressing mixed opinions on patient care impacts and privacy concerns
Bokolo et al. [29]	Mental Disease	Depression detection from Twitter	RoBERTa, DeBERTa	632,000 tweets	97.48%	Transformer models like RoBERTa excel in depression detection from Twitter data, outperforming traditional ML approaches
Crasto et al. [59]	Mental Disease	Mental health interventions using CAs supported by LLMs	DialoGPT	Counselchat (includes tags of illness); question answers from 100 college students	N/A	The DialoGPT model, demonstrating higher perplexity and preferred by 63% of college participants for its human-like and empathetic responses, was chosen as the most suitable system for addressing student mental health issues
Dai et al. [24]	Mental Disease	Psychiatric patient screening	BERT, DistilBERT, ALBERT, ROBERTa	500 EHRs	Accuracy not reported, F1 0.830	BERT models, especially with feature dependency, effectively classify psychiatric conditions from EHRs
Danner et al. [60]	Mental Disease	Detecting depression using LLMs through clinical interviews	BERT; GPT 3.5, ChatGPT 4	DAIC-WOZ; Extended-DAIC; simulated data	78%	The study assessed the abilities of GPT-3.5-turbo and ChatGPT-4 on the DAIC-WOZ dataset, which yielded F1 scores of 0.78 and 0.61, respectively, and a custom BERT model, extended-trained on a larger dataset, which achieved an F1 score of 0.82 on the Extended-DAIC dataset, in recognizing depression in text
Dergaa et al. [61]	Mental Disease	Simulated mental health assessments and interventions with ChatGPT	GPT 3.5	Fictional patient data; 3 scenarios	N/A	ChatGPT showed limitations in complex medical scenarios, underlining its unpreparedness for standalone use in mental health practice
Diniz et al. [62]	Mental Disease	Detecting suicidal ideation using LLMs through Twitter texts	BERT model for Portuguese, Multilingual BERT (base), BERTimbau	Non-clinical texts from tweets (user posts of the online social network Twitter)	95%	The Boamente system demonstrated effective text analysis for suicidal ideation with high privacy standards and actionable insights for mental health professionals. The best-performing BERTimbau Large model (accuracy: 0.955; precision: 0.961; F-score: 0.954; AUC: 0.954) significantly excelled in detecting suicidal tendencies, showcasing robust accuracy and recall in model evaluations
D’Souza et al. [27]	Mental Disease	Responding to psychiatric case vignettes with diagnostic and management strategies	GPT 3.5	Fictional patient data from clinical case vignettes; 100 cases	61%	ChatGPT 3.5 showed high competence in handling psychiatric case vignettes, with strong diagnostic and management strategy generation
Elyoseph et al. [63]	Mental Disease	Evaluating emotional awareness compared to general population norms	GPT 3.5	Fictional scenarios from the LEAS; 750 participants	85%	ChatGPT showed higher emotional awareness compared to the general population and improved over time
Elyoseph et al. [64]	Mental Disease	Assessing suicide risk in fictional scenarios and comparing to professional evaluations	GPT 3.5	Fictional patient data; text vignettescompared to 379 professionals	N/A	ChatGPT underestimated suicide risks compared to mental health professionals, indicating the need for human judgment in complex assessments
Elyoseph et al. [30]	Mental Disease	Evaluating prognosis in depression compared to other LLMs and professionals	GPT 3.5, GPT 4	Fictional patient data; text vignettescompared to 379 professionals	N/A	ChatGPT 3.5 showed a more pessimistic prognosis in depression compared to other LLMs and mental health professionals
Esackimuthu et al. [65]	Mental Disease	Depression detection from social media text	ALBERT base v1	ALBERT base v1 data	50%	ALBERT shows potential in detecting depression signs from social media texts but faces challenges due to complex human emotions
Farhat et al. [66]	Mental Disease	Evaluation of ChatGPT as a complementary mental health resource	ChatGPT	Responses generated by ChatGPT	N/A	ChatGPT displayed significant inconsistencies and low reliability when providing mental health support for anxiety and depression, underlining the necessity of validation by medical professionals and cautious use in mental health contexts
Farruque et al. [67]	Mental Disease	Depression level detection modeling	Mental BERT (MBERT)	13,387 Reddit samples	Accuracy not reported, F1 0.81	MBERT enhanced with text excerpts significantly improves depression level classification from social media posts
Farruque et al. [68]	Mental Disease	Depression symptoms modeling from Twitter	BERT, Mental-BERT	6077 tweets and 1500 annotated tweets	Accuracy not reported, F1 0.45	Semi-supervised learning models, iteratively refined with Twitter data, improve depression symptom detection accuracy
Friedman and Ballentine [69]	Mental Disease	Evaluation of LLMs in data-driven discovery: correlating sentiment changes with psychoactive experiences	BERTowid, BERTiment	Erowid testimonials; drug receptor affinities; brain gene expression data; 58K annotated Reddit posts	N/A	This paper found that LLM methods can create unified and robust quantifications of subjective experiences across various psychoactive substances and timescales. The representations learned are evocative and mutually confirmatory, indicating significant potential for LLMs in characterizing psychoactivity
Ghanadian et al. [70]	Mental Disease	Suicidal ideation detection using LLMs through social media texts	ALBERT, DistilBERT, ChatGPT, Flan-T5, Llama	UMD Dataset; Synthetic Datasets (Generated using LLMs like Flan-T5 and Llama2, these datasets augment the UMD dataset to enhance model performance)	87%	The synthetic data-driven method achieved consistent F1-scores of 0.82, comparable to real-world data models yielding F1-scores between 0.75 and 0.87. When 30% of the real-world UMD dataset was combined with the synthetic data, the performance significantly improved, reaching an F1-score of 0.88 on the UMD test set. This result highlights the effectiveness of synthetic data in addressing data scarcity and enhancing model performance
Hadar- Shoval et al. [71]	Mental Disease	Differentiating emotional responses in BPD and SPD scenarios using mentalising abilities	GPT 3.5	Fictional patient data (BPD and SPD scenarios); AI-generated data	N/A	ChatGPT effectively differentiated emotional responses in BPD and SPD scenarios, showing tailored mentalizing abilities
Hayati et al. [72]	Mental Disease	Detecting depression by Malay dialect speech using LLMs	GPT 3	Interviews with 53 adults fluent in Kuala Lumpur (KL), Pahang, or Terengganu Malay dialects	73%	GPT-3 was tested on three different dialectal Malay datasets (combined, KL, and non-KL). It performed best on the KL dataset with a max_example value of 10, which achieved the highest overall performance. Despite the promising results, the non-KL dataset showed the lowest performance, suggesting that larger or more homogeneous datasets might be necessary for improved accuracy in depression detection tasks
He et al. [73]	Mental Disease	Evaluation of CAs handling counseling for people with autism supported by LLMs	ChatGPT	Public available data from the web-based medical consultation platform DXY	N/A	The study found that 46.86% of assessors preferred responses from physicians, 34.87% favored ChatGPT, and 18.27% favored ERNIE Bot. Physicians and ChatGPT showed higher accuracy and usefulness compared to ERNIE Bot, while ChatGPT outperformed both in empathy. The study concluded that while physicians’ responses were generally superior, LLMs like ChatGPT can provide valuable guidance and greater empathy, though further optimization and research are needed for clinical integration
Hegde et al. [74]	Mental Disease	Depression detection using supervised learning	Ensemble of ML classifiers, BERT	Social media text data	Accuracy not reported, F1 0.479	BERT-based Transfer Learning model outperforms traditional ML classifiers in detecting depression from social media texts
Heston T.F. et al. [75]	Mental Disease	Simulating depression scenarios and evaluating AI’s responses	GPT 3.5	Fictional patient data; 25 conversational agents	N/A	ChatGPT-3.5 conversational agents recommended human support at critical points, highlighting the need for AI safety in mental health
Hond et al. [76]	Mental Disease	Early depression risk detection in cancer patients	BERT	16,159 cancer patients’ EHR data	Accuracy not reported, AUROC 0.74	Machine learning models predict depression risk in cancer patients using EHRs, with structured data models performing best
Howard et al. [77]	Mental Disease	Detecting suicidal ideation using LLMs through social media texts	DeepMoji, Universal Sentence Encoder, GPT 1	1588 labeled posts from the Computational Linguistics and Clinical Psychology 2017 shared task	Accuracy not reported, F1 0.414	The top-performing system, utilizing features derived from the GPT-1 model fine-tuned on over 150,000 unlabeled Reachout.com posts, achieved a new state-of-the-art macro-averaged F1 score of 0.572 on the CLPsych 2017 task without relying on metadata or preceding posts. However, error analysis indicated that this system frequently misses expressions of hopelessness
Hwang et al. [78]	Mental Disease	Generating psychodynamic formulations in psychiatry based on patient history	GPT 4	Fictional patient data from published psychoanalytic literature; 1 detailed case	N/A	GPT-4 successfully created relevant and accurate psychodynamic formulations based on patient history
Ilias et al. [79]	Mental Disease	Stress and depression identification in social media	BERT, MentalBERT	Public datasets	Accuracy not reported, F1 0.73	Extra-linguistic features improve calibration and performance of models in detecting stress and depression from texts
Janatdoust et al. [80]	Mental Disease	Depression signs detection from social media text	Ensemble of BERT, ALBERT, DistilBERT, RoBERTa	16,632 social media comments	61%	Ensemble models effectively classify depression signs from social media, utilizing multiple language models for improved accuracy
Kabir et al. [81]	Mental Disease	Depression severity detection from tweets	BERT, DistilBERT	40,191 tweets	Accuracy not reported, AUROC 0.74–0.86	Models effectively classify social media texts into depression severity categories, with high confidence and accuracy
Kumar et al. [82]	Mental Disease	Evaluation of GPT 3 in mental health intervention	GPT 3	209 participants responses, with 189 valid responses after filtering	N/A	This paper found that interaction with either of the chatbots improved participants’ intent to practice mindfulness again, while the tutorial video enhanced their overall experience of the exercise. These findings highlighted the potential promise and outlined directions for exploring the use of LLM-based chatbots for awareness-related interventions
Lam et al. [83]	Mental Disease	Multi-modal depression detection	Transformer, 1D CNN	189 DAIC-WOZ participants	Accuracy not reported, F1 0.87	Multi-modal models combining text and audio data effectively detect depression, enhanced by data augmentation
Lau et al. [28]	Mental Disease	Depression severity assessment	Prefix-tuned LLM	189 clinical interview transcripts	Accuracy not reported, RMSE 4.67	LLMs with prefix-tuning significantly enhance depression severity assessment, surpassing traditional methods
Levkovich and Elyoseph [31]	Mental Disease	Diagnosing and treating depression, comparing GPT models with primary care physicians	GPT 3.5, GPT 4	Fictional patient data from clinical case vignettes; repeated multiple times for consistency	N/A	ChatGPT aligned with guidelines for depression management, contrasting with primary care physicians and showing no gender or socioeconomic biases
Levkovich and Elyoseph [32]	Mental Disease	Evaluating suicide risk assessments by GPT models and mental health professionals	GPT 3.5, GPT 4	Fictional patient data; text vignettes compared to 379 professionals	N/A	GPT 4’s evaluations of suicide risk were similar to mental health professionals, though with some overestimations and underestimations
Li et al. [84]	Mental Disease	Evaluating performance on psychiatric licensing exams and diagnostics	GPT 4, Bard and Llama-2	Fictional patient data in exam and clinical scenario questions; 24 experienced psychiatrists	69%	GPT 4 outperformed other models in psychiatric diagnostics, closely matching the capabilities of human psychiatrists
Liyanage et al. [85]	Mental Disease	Data augmentation for wellness dimension classification in Reddit posts	GPT 3.5	Real patient data from Reddit posts; 3092 instances, post-augmentation 4376 records	69%	ChatGPT models effectively augmented Reddit post data, significantly improving classification performance for wellness dimensions
Lossio-Ventura et al. [26]	Mental Disease	Evaluations of LLMs for sentiment analysis through social media texts	ChatGPT;Open Pre-Trained Transformers (OPT)	NIH Data Set; Stanford Data Set	86%	This paper revealed high variability and disagreement among sentiment analysis tools when applied to health-related survey data. OPT and ChatGPT demonstrated superior performance, outperforming all other tools. Moreover, ChatGPT outperformed OPT, achieving a 6% higher accuracy and a 4% to 7% higher F-measure
Lu et al. [86]	Mental Disease	Depression detection via conversation turn classification	BERT, transformer encoder	DAIC dataset	Accuracy not reported, F1 0.75	Novel deep learning framework enhances depression detection from psychiatric interview data, improving interpretability
Ma et al. [87]	Mental Disease	Evaluation of mental health intervention CAs supported by LLMs	GPT 3	120 Reddit posts (2913 user comments)	N/A	The study highlighted that CAs like Replika, powered by LLMs, offered crucial mental health support by providing immediate, unbiased assistance and fostering self-discovery. However, they struggled with content filtering, consistency, user dependency, and social stigma, underscoring the importance of cautious use and improvement in mental wellness applications
Mazumdar et al. [88]	Mental Disease	Classifying mental health disorders and generating explanations	GPT 3, BERT-large, MentalBERT, ClinicBERT, and PsychBERT	Real patient data sourced from Reddit posts	87%	GPT 3 outperformed other models in classifying mental health disorders and generating explanations, showing promise for AI-IoMT deployment
Metzler et al. [89]	Mental Disease	Detecting suicidal ideation using LLMs through Twitter texts	BERT, XLNet	3202 English tweets	88.50%	BERT achieved F1-scores of 0.93 for accurately labeling tweets as about suicide and 0.74 for off-topic tweets in the binary classification task. Its performance was similar to or exceeded human performance and matched that of state-of-the-art models on similar tasks
Owen et al. [90]	Mental Disease	Depression signal detection in Reddit posts	BERT, MentalBERT	Reddit datasets	Accuracy not reported, F1 0.64	Effective identification of depressive signals in online forums, with potential for early intervention
Parker et al. [91]	Mental Disease	Providing information on bipolar disorder and generating creative content	GPT 3	N/A	N/A	GPT-3 provided basic material on bipolar disorders and creative song generation, but lacked depth for scientific review
Perlis et al. [34]	Mental Disease	Evaluation of GPT 4 for clinical decision support in bipolar depression	GPT 4 turbo (gpt-4-1106-preview)	Recommendations generated by the augmented GPT-4 model and responses from clinicians treating bipolar disorder	50.80%	This paper found that the augmented GPT 4 model had a Cohen’s kappa of 0.31 with expert consensus, identifying the optimal treatment in 50.8% of cases and placing it in the top 3 in 84.4% of cases. In contrast, the base model had a Cohen’s kappa of 0.09 and identified the optimal treatment in 23.4% of cases, highlighting the enhanced performance of the augmented model in aligning with expert recommendations
Poświata and Perełkiewicz [92]	Mental Disease	Depression sign detection using RoBERTa	RoBERTa, DepRoBERTa	RoBERTa models’ data	Accuracy not reported, F1 0.583	RoBERTa and DepRoBERTa ensemble excels in classifying depression signs, securing top performance in a competitive environment
Pourkeyvan et al. [25]	Mental Disease	Mental health disorder prediction from Twitter	BERT models from Hugging Face	11,890,632 tweets and 553 bio-descriptions	97%	Superior detection of depression symptoms from social media, demonstrating the efficacy of advanced NLP models
Sadeghi et al. [93]	Mental Disease	Detecting depression using LLMs through interviews	GPT 3.5-Turbo, RoBERTa	E-DAIC (219 participants)	N/A	The study achieved its lowest error rates, a Mean Absolute Error (MAE) of 3.65 on the dev set and 4.26 on the test set, by fine-tuning DepRoBERTa with a specific prompt, outperforming manual methods and highlighting the potential of automated text analysis for depression detection
Schubert et al. [94]	Mental Disease	Evaluation of LLMs’ performance on neurology board-style examinations	ChatGPT 3.5, ChatGPT 4.0	A question bank from an educational company with 2036 questions that resemble neurology board questions	85%	ChatGPT 4.0 excelled over ChatGPT 3.5, achieving 85.0% accuracy versus 66.8%. It surpassed human performance in specific areas and exhibited high confidence in responses. Longer questions tended to result in more incorrect answers for both models
Senn et al. [95]	Mental Disease	Depression classification from interviews	BERT, RoBERTa, DistilBERT	189 clinical interviews	Accuracy not reported, F1 0.93	Ensembles of BERT models enhance depression detection robustness in clinical interviews
Singh and Motlicek [96]	Mental Disease	Depression level classification using BERT, RoBERTa, XLNet	Ensemble of BERT, RoBERTa, XLNet	Ensemble of models	54%	Ensemble model accurately classifies depression levels from social media text, ranking highly in competitive settings
Sivamanikandan S. et al. [97]	Mental Disease	Depression level classification	DistilBERT, RoBERTa, ALBERT	Social media posts	Accuracy not reported, F1 0.457	Transformer models classify depression levels effectively, with RoBERTa achieving the best performance
Spallek et al. [98]	Mental Disease	Providing educational material on mental health and substance use	GPT-4	Real-world queries from mental health and substance use portals; 10 queries	N/A	GPT 4’s outputs were substandard compared to expert materials in terms of depth and adherence to communication guidelines
Stigall et al. [99]	Mental Disease	Emotion classification using LLMs through social media texts	EmoBERTTiny	A collection of publicly available datasets hosted on Kaggle and Huggingface	93.14% (sentiment analysis), 85.46% (emotion analysis)	EmoBERTTiny outperformed pre-trained and state-of-the-art models in all metrics and computational efficiency, achieving 93.14% accuracy in sentiment analysis and 85.48% in emotion classification. It processes a 256-token context window in 8.04 ms post-tokenization and 154.23 ms total processing speed
Suri et al. [100]	Mental Disease	Depressive tendencies detection using multimodal data	BERT	5997 tweets	97%	Multimodal BERT frameworks significantly enhance detection of depressive tendencies from complex social media data
Tao et al. [101]	Mental Disease	Detecting anxiety and depression using LLMs through dialogs in real-life scenarios	ChatGPT	Speech data from nine Q&A tasks related to daily activities (75 patients with anxiety and 64 patients with depression)	67.62%	This paper introduced a virtual interaction framework using LLMs to mitigate negative psychological states. Analysis of Q&A dialogs demonstrated ChatGPT’s potential in identifying depression and anxiety. To enhance classification, four language features, including prosodic and speech rate, positively impacted classification
Tey et al. [102]	Mental Disease	Pre- and post-depressive detection from tweets	BERT, supplemented with emoji decoding	Over 3.5 million tweets	Accuracy not reported, F1 0.90	Augmented BERT model classifies Twitter users into depressive categories, enhancing early depression detection
Toto et al. [103]	Mental Disease	Depression screening using audio and text	AudiBERT	189 clinical interviews	Accuracy not reported, F1 0.92	AudiBERT outperforms traditional and hybrid models in depression screening, utilizing multimodal data
Vajre et al. [104]	Mental Disease	Detecting mental health using LLMs through social media texts	PsychBERT	Twitter hashtags and Subreddit (6 domains: anxiety, mental health, suicide, etc)	Accuracy not reported, F1 0.63	The study identified PsychBERT as the highest-performing model, achieving an F1 score of 0.98 in a binary classification task and 0.63 in a more challenging multi-class classification task, indicating its superiority in handling complex mental health-related data. Additionally, PsychBERT’s explainability was enhanced by using the Captum library, which confirmed its ability to accurately identify key phrases indicative of mental health issues
Verma et al. [105]	Mental Disease	Detecting depression using LLMs through textual data	RoBERTa	Mental health corpus	96.86%	The study successfully used a RoBERTa-base model to detect depression with a high accuracy of 96.86%, showcasing the potential of AI in identifying mental health issues through linguistic analysis
Wan et al. [106]	Mental Disease	Family history identification in mood disorders	BERT–CNN	12,006 admission notes	97%	High accuracy in identifying family psychiatric history from EHRs, suggesting utility in understanding mood disorders
Wang et al. [107]	Mental Disease	Enhancing depression diagnosis and treatment through the use of LLMs	LLaMA-7B; ChatGLM-6B, Alpaca, LLMs + Knowledge	Chinese Incremental Pre-training Dataset	N/A	The study assessed LLMs’ performance in mental health, emphasizing safety, usability, and fluency and integrating mental health knowledge to improve model effectiveness, enabling more tailored dialogs for treatment while ensuring safety and usability
Wang et al. [33]	Mental Disease	Detecting depression using LLMs through microblogs	BERT, RoBERTa, XLNet	13,993 microblogs collected from the Sina Weibo	Accuracy not reported, F1 0.424	RoBERTa achieved the highest macro-averaged F1 score of 0.424 for depression classification, while BERT scored the highest micro-averaged F1 score of 0.856. Pretraining on an in-domain corpus improved model performance
Wei et al. [108]	Mental Disease	Evaluation of ChatGPT in psychiatry	ChatGPT	Theoretical analysis and literature reviews	N/A	The paper found ChatGPT useful in psychiatry, stressing ethical use and human oversight, while noting challenges in accuracy and bias, positioning AI as a supportive tool in care
Wu et al. [109]	Mental Disease	Expanding dataset of Post-Traumatic Stress Disorder (PTSD) using LLMs	GPT 3.5 Turbo	E-DAIC (219 participants)	Accuracy not reported, F1 0.63	This paper demonstrated that two novel text augmentation frameworks using LLMs significantly improved PTSD diagnosis by addressing data imbalances in NLP tasks. The zero-shot approach, which generated new standardized transcripts, achieved the highest performance improvements, while the few-shot approach, which rephrased existing training samples, also surpassed the original dataset’s efficacy
Yongsatianchot et al. [110]	Mental Disease	Evaluation of LLMs’ perception of emotion	Text-davinci-003, ChatGPT, GPT 4	Responses from three OpenAI LLMs to the Stress and Coping Process Questionnaire	N/A	The study applied the SCPQ to three OpenAI LLMs—davinci-003, ChatGPT, and GPT 4—and found that while their responses aligned with human dynamics of appraisal and coping, they did not vary across key appraisal dimensions as predicted and differed significantly in response magnitude. Notably, all models reacted more negatively than humans to negative scenarios, potentially influenced by their training processes
Zhang et al. [111]	Mental Disease	Detecting depression trends using LLMs through Twitter texts	RoBERTa, XLNet	2575 Twitter users with depression identified via tweets and profiles	78.90%	This study developed a fusion model that accurately classified depression among Twitter users with 78.9% accuracy. It identified key linguistic and behavioral indicators of depression and demonstrated that depressive users responded to the pandemic later than controls. The findings suggest the model’s effectiveness in noninvasively monitoring mental health trends during major events like COVID-19
